# Every Young Athlete Counts: Are Tailored Doping Prevention Programs Necessary in Young Elite Sports?

**DOI:** 10.3389/fspor.2022.858730

**Published:** 2022-05-27

**Authors:** Katharina Pöppel, Dirk Büsch

**Affiliations:** Institute of Sport Science, Carl von Ossietzky University of Oldenburg, Oldenburg, Germany

**Keywords:** anti-doping education, young elite athletes, individualization, evaluation, gender

## Abstract

Conclusions from doping prevention literature recommend tailored anti-doping education for athletes' specific needs. Newer approaches like the International Standard for Education of the World Anti-Doping Agency recommend a needs assessment before implementing measures. The International Standard for Education refers to the type of sports and its associated risk for doping. Following this idea, elite athletes from different types of sports should differ in their prerequisites for doping prevention. Consequently, the guiding research question focused on exploring the doping-prevention-related background of young athletes as a particular group for prevention efforts. Sixty young elite athletes (58.3% male) took part in a cross-sectional online survey, which was quantitatively analyzed. Participants included 26 athletes from a sport with low doping prevalence (sailing) and 34 athletes from a sport associated with high doping prevalence (wrestling). Sailors and wrestlers differed concerning the perceived resistance against doping temptations (*p* = 0.031, *r* = 0.31) and the estimated actual doping prevalence regarding sports in general (national frame: *p* < 0.001, *r* = 0.60; international frame: *p* = 0.013, *r* = 0.43). No differences between the two types of sports occurred, referring to doping attitudes, tendency to disengage morally, or topics athletes wish to learn about during doping prevention measures. All results indicated a good baseline for doping prevention with young elite athletes at the beginning of their careers. There is no sport-specific needs profile that could be used as a base for tailored measures. However, the data suggest that a differentiated consideration of gender could be helpful in the planning of doping prevention measures.

## Introduction

Peak performance is appreciated inside and outside elite sports. For this reason, some athletes try to gain an advantage by prohibited means, commonly known as doping. Highlighting the complexity, Petróczi(2021, p. S16) describes the phenomenon as follows: “When strategies for boosting performance employ substances or methods specifically outlawed by a governing body, such as the World Anti-Doping Agency (WADA), the practices become doping.” Besides outlawing doping offenses, WADA aims to prevent doping by developing and implementing doping prevention measures (World Anti-Doping Agency, 2021b). Therefore, athletes should be qualified to make informed decisions as part of the management of their sports performance. Understanding doping prevention as a learning process, this brief research report aims to specify a natural baseline level of characteristic variables of young athletes involved in talent development programs of different types of sports prior to doping prevention.

Doping prevention aims to support athletes to refrain from doping and arrange a fair and clean sports environment. Different prevention approaches were published recently to support athletes in resisting doping and maintaining the spirit of sport. For example, WADA's International Standard for Education (ISE, World Anti-Doping Agency, 2021b) pictures the shift from deterrence-based (e.g., Goldberg et al., 1991) to skill-oriented and values-based doping prevention or the protection of clean athletes (Petróczi et al., 2021). The ISE refers to a multifaceted handling of doping prevention: It strives for an early and global implementation of anti-doping education, offers constructive handling of doping prevention, and introduces ideas for interactive learning in the cognitive and affective domain, including values-based education. The ISE aims to enable a tailored application built upon an evaluation of the general setting and an additional needs assessment. The needs assessment should consider the specifics of sports, participants' learning needs as “a good first step to planning education,” and the risk for doping in different types of sport (World Anti-Doping Agency, 2021b, p. 34). Even before the publication of the ISE, ideas for increasing the individualization of doping prevention measures have emerged.

Models of doping behavior guide the understanding of athletes' behavior and influence how prevention is implemented (Hauw and McNamee, 2015). Numerous publications on doping and prevention refer to the theory of planned behavior (Ajzen, 1991; Pöppel, 2021). They emphasize the impact of positive doping attitudes and perceived social norms as correlates of doping intentions and behavior or extend the theoretical model by adding the use of supplements or self-efficacy to resist doping (Ntoumanis et al., 2014). However, the focus on this theory is critically discussed (Petróczi et al., 2017). Nevertheless, many doping prevention studies use these variables to evaluate the effectiveness of interventions (Pöppel, 2021). Mainly newer approaches highlight the importance of ethics, moral disengagement, or resistance against doping temptations as target variables and broaden the prevention perspective (Elbe and Brand, 2016; Hurst et al., 2020; Kavussanu et al., 2021). According to these studies, doping attitudes, moral disengagement, and self-regulatory effectiveness represent variables of interest in the context of doping prevention. Irrespective of the concrete configuration of doping prevention, researchers increasingly demand scientific monitoring and evaluation of these measures to understand better the effect (Boardley et al., 2021; Pöppel, 2021).

Recommendations for doping prevention tend to be literature-based (e.g., Backhouse et al., 2012), developed by expert consensus (Boardley et al., 2021; Petróczi et al., 2021), or based on conclusions from empirical data (e.g., Elbe and Brand, 2016). These recommendations involve individualizing prevention measures, including online options, protecting clean athletes, and empowering informed decision-making (e.g., Backhouse et al., 2012; Pöppel, 2021). Hence, the request for tailored approaches includes the assumption that individual requirements of athletes exist. In 2017, for example, the German Nationale Anti-Doping Agentur (2020) launched the *Together Against Doping* program (German: *Gemeinsam gegen Doping*), which offers athletes a choice of different thematic units as part of a modular system. The content areas range from basic questions about doping to optimizing performance through nutrition. The idea of tailored approaches corresponds to the ISE's ideas of a prior needs assessment and the focus on target groups with a high risk for doping, which should be prioritized in education (World Anti-Doping Agency, 2021b). Therefore, this study aims to conduct a baseline level of variables characteristic of potential doping behavior and relevant to doping prevention.

Following official laboratory data, doping appears to be a comparatively rare phenomenon. Thus, World Anti-Doping Agency (2021a) reported a doping prevalence of 0.7% for sports overall. The prevalence considering Olympic sports ranged from 0% in sailing to 1.2% in weightlifting. However, the number of athletes using prohibited aids seems more comprehensive. Presumably, we face many unreported cases and difficulties in specifying an approximate value (Gleaves et al., 2021). According to their review data, the reported prevalence rates ranged from 0 to 73%. Facing the immense interval, the authors criticized a weak database of the underlying studies and referred to specifics of the population examined, like sports, gender, or geographic differences. While laboratory data underestimate the actual doping prevalence, individuals inside and outside elite sports assume that sports are more polluted than these official data indicate. Additionally, we face a more optimistic view of the situations in one's country. Coaches or fans, for example, estimated the prevalence in international competitive sports to be significantly higher than in the national setting (Solberg et al., 2010; Pöppel and Büsch, 2019).

A suspicion of doping can have severe consequences for athletes, including a competition ban or a withdrawal of achievements (World Anti-Doping Agency, 2021c). Therefore, athletes who engage in doping generally avoid doping-related disclosures. One can assume that response bias, like social desirability, influences athletes' answers concerning doping behavior and associated variables (Gucciardi et al., 2010; Petróczi and Nepusz, 2011). For this reason, researchers recommend a further application of indirect measures (Petróczi, 2016).

Gatterer et al. (2021) concluded that almost 75% of young elite athletes had received anti-doping education prior to the publication of the ISE (World Anti-Doping Agency, 2021b). These athletes rated the trust in the measures and the usefulness of this education as good. Considering that confidence in the fight against doping is decreased among older elite athletes due to negative experiences in doping controls and thus their socialization in elite sports (Petróczi et al., 2021), this good starting position should be used. Despite attempts to achieve international standardization, geographical differences concerning doping knowledge, beliefs, and attitudes can be found in young elite athletes (Königstein et al., 2021). The authors highlight the existence of a sound knowledge base as a valuable basis for doping prevention, influencing other variables such as attitudes or beliefs. A sound knowledge might provide direction, also in the gray areas as the borderline between prohibited and not prohibited substance use is blurred.

Doping prevention should start early, like in preadolescence, and adolescent athletes are a favorite target group as they still develop their understanding of sports and enable most likely a primary prevention approach (Nicholls et al., 2017; Königstein et al., 2021). Additionally, we face an early onset of doping behavior as even under-10-years-olds reported doping (Nicholls et al., 2017). It is to assume that this age group is not aware of the consequences of harmful behavior yet and needs support to reflect on doping. Therefore, it is even more crucial to constructively equip young elite athletes for informed-decision making.

To gain a deeper insight, participants in this study included athletes with a high competition level, as this level corresponds to a high pressure to perform and a natural confrontation with doping topics. Furthermore, athletes should compete in types of sports which represent *extreme groups* concerning the risk for doping based on the respective doping prevalence according to World Anti-Doping Agency (2021a), which encompasses all elite athletes from junior to senior level. Referring to extreme groups might enhance the probability of differences in athletes' needs. Additionally, preferably young athletes should be included, as they have a favorable position for doping prevention (e.g., Backhouse et al., 2012; Königstein et al., 2021).

## Research Questions

From a scientific perspective, recommendations for doping prevention include tailored measures for specific groups and an early onset (e.g., Backhouse et al., 2012). They rather represent a top-down position based on theoretical deductions (Boardley et al., 2021; Petróczi et al., 2021; Pöppel, 2021). An empirical needs assessment of the respective target groups prior to doping prevention is lacking. According to the idea of tailored doping prevention, differences should appear regarding the athlete's prerequisites and needs (e.g., depending on the different types of sports). The article is guided by the research question: How is the doping prevention-related background constituted in young elite athletes? And more specifically: Are sport-specific differences already apparent at the beginning of a career in the highest performance level in sports? In contrast to elite sports in general, it covers specifically the entrance in the high-performance level.

Approaches on the evaluation of anti-doping education represent a retrospective view (e.g., Hurst et al., 2020; Gatterer et al., 2021). This research report takes a forward-looking perspective and aims to provide an empirical baseline of young elite athletes' prerequisites concerning upcoming doping prevention. The analysis is based on variables discussed in the literature as relevant. Athletes should differ concerning:

1. the perceptions of the extent to which doping is prevalent in sport (cf. Pöppel and Büsch, 2019);
2. their doping attitudes (cf. Elbe and Brand, 2016);
3. their willingness to morally disengage (cf. Kavussanu et al., 2021);
4. their resistance against doping temptations (cf. Kavussanu et al., 2021);
5. their content-related requests for doping prevention (cf. World Anti-Doping Agency, 2021b).

## Methods

### Study Design, Sample, and Recruitment

To answer the research questions, a cross-sectional online survey (survey tool: LimeSurvey) was conducted. The authors addressed extreme groups of athletes according to World Anti-Doping Agency's (2021a) report on doping prevalence to compare athletes with a comparable competition level but different sport-specific socialization. As representatives of the extreme groups for Olympic sports, this study addressed young athletes involved in talent development programs from sailing (*low* doping prevalence: 0%) and wrestling (*high* doping prevalence: 1.1%). They were included in this study representing young elite sports. The sports directors coordinated the data assessment within their federation by obtaining informed consent of the participants or their parents in case of minors and disseminating the invitation and the link to participate in the survey. The study was conducted following the recommendations of the Carl von Ossietzky University of Oldenburg, Germany, and the local committee approved the protocol for research assessment and ethics. All subjects gave their written informed consent in accordance with the Declaration of Helsinki. Data assessment took place in March 2020 (sailing) and September 2021 (wrestling).

### Procedure

The online survey was primarily based on existing and validated questionnaires applied in a German translation. The athlete's attitude toward doping was assessed by the short version of the Performance Enhancement Attitude Scale (PEAS-S; Vargo et al., 2015). The scale indicated adequate reliability for a short form (Cronbach's α = 0.72, Widaman et al., 2011). Participants indicated their agreement on a 6-point Likert scale (1 = strongly disagree to 6 = strongly agree), which led to a sum value (scores 8–14: strongly disagree, 15–21: disagree, 22–28: slightly disagree, 29–35: slightly agree, 36–42: agree, 43–48: strongly agree). High values indicated a more lenient doping attitude. Their moral perspective was assessed by the short version of the Doping Moral Disengagement Scale (DMDS-S; 7-point Likert scale from 1 = strongly disagree to 7 = strongly agree; Boardley et al., 2018). Its psychometric properties can be rated as weak in the underlying sample (Cronbach's α = 0.60, Widaman et al., 2011). Additionally, the athlete's resistance against doping was evaluated based on the Doping Self-Regulatory Efficacy Scale (DSRES; Boardley et al., 2018). The participants indicated their confidence to resist on a 5-point Likert scale from 1 (no confidence) to 5 (complete confidence). According to Widaman et al. (2011), the scale's reliability is acceptable (Cronbach's α = 0.89). DMDS-S and DSRES were interpreted based on the mean. Higher values corresponded to a critical doping representation.

The three short questionnaires were embedded by estimations of the actual national and international doping prevalence at the beginning of the survey, and a prioritization of topics of the doping prevention program *Together Against Doping* of the German Nationale Anti-Doping Agentur (2020), as well as questions concerning the athlete's supplement use at the end of the survey. Finally, participants conveyed demographic data, including age, gender, squad status, and information concerning doping, doping prevention, and supplements.

### Analysis

Data were analyzed applying the following software packages: IBM SPSS Statistics 27; JASP statistic software, version 0.16 (JASP Team, 2021); as well as G^*^Power, version 3.1.9.7 (Faul et al., 2020) to perform sensitivity analyses. Shapiro-Wilk tests carried out in advance showed a deviation from normality for the estimation of the doping prevalence, doping attitudes, moral disengagement, and self-regulatory efficacy. For this reason, non-parametric tests were applied to compare the two types of sport and a subsequent exploratory data analysis comparing male and female participants. As the logic of the different tests is based on rank data, medians (*Mdn*) and median absolute deviations (*MAD*) were reported in addition to means (*M*) and standard deviations (*SD*).

## Results

Altogether 60 athletes representing the highest levels of elite sports in Germany (*n* = 34 wrestling, *n* = 26 sailing) completed the survey, whereby two participants skipped conveying demographic information. The participant's mean age was 18.14 years (*SD* = 2.24), and 58.2 % (*n* = 35) of the sample was male (see Table 1). The participants of the two types of sports did not differ concerning age [*t*_(56)_ = 0.63, *p* = 0.529] or previous experience concerning doping prevention measures [*t*_(56)_ = 1.28, *p* = 0.206]. Most participants (68.3 %, *n* = 39) took part in one or two doping prevention measures. Thus, the baseline level evaluated here was heterogenous concerning prior prevention experience. Sixty percent of the participants (*n* = 36) indicated that they did not search for information on doping themselves.

**Table 1:** Characteristics of the sample.

		**Sailing (*n* = 26)**	**Wrestling (*n* = 34)**	**Overall (*N* = 60)**
Gender	Male Female	42.3 % (*n* = 11) 57.7 % (*n* = 15)	70.7 % (*n* = 24) 23.5 % (*n* = 8)	58.3 % (*n* = 35) 38.3 % (*n* = 23)
Age (years)	*M* (*SD*) Range	18.35 (1.36) 15–20	17.97 (2.78) 15–30	18.14 (2.24) 15–30
Squad	Perspective squad Youth squad 1 Youth squad 2 Federal state squad	73.1 % (*n* = 19) 11.5 % (*n* = 3) 15.4 % (*n* = 4)	14.7 % (*n* = 5) 32.4 % (*n* = 11) 47.1 % (*n* = 16)	8.3 % (*n* = 5) 5% (*n* = 30) 31.7 % (*n* = 19) 6.7 % (*n* = 4)
Style	Single-handed	42.3 % (*n* = 11)		
	Double-handed	57.7 % (*n* = 15)		
	Greco-roman		6.7 % (*n* = 4)	
	Freestyle		46.7 % (*n* = 28)	
Participation in doping prevention measures	0 1 2 3 4 5 More than 5	23.1% (*n* = 6) 34.6% (*n* = 9) 1% (*n* = 4) 15.4% (*n* = 4) 3.8% (*n* = 1) 7.7% (*n* = 2)	38.2% (*n* = 13) 17.6% (*n* = 6) 26.5% (*n* = 9) 8.8% (*n* = 3) 2.9% (*n* = 1)	31.7% (*n* = 19) 25.0% (*n* = 15) 21.7% (*n* = 13) 11.7% (*n* = 7) 1.7% (*n* = 1) 5.0% (*n* = 3)
Own search for doping information	Yes No	53.8% (*n* = 14) 46.2% (*n* = 12)	23.5% (*n* = 8) 70.6% (*n* = 24)	36.7% (*n* = 22) 60.0% (*n* = 36)
Supplements use	Yes No	34.6% (*n* = 9) 65.4% (*n* = 17)	26.5% (*n* = 9) 67.6% (*n* = 23)	30.0% (*n* = 18) 66.7% (*n* = 40)
Application Cologne list	Yes No	61.5% (*n* = 16) 38.4% (*n* = 10)	61.8% (*n* = 21) 29.4% (*n* = 10)	61.7% (*n* = 37) 33.3% (*n* = 20)

*Perspective squad: former B- and C-squad for athletes with outstanding performance perspective, second highest squad; youth squad 1: third highest squad, youth squad 2: fourth highest squad, federal state squad: lowest national squad level according to the German Olympic Sports Confederation*.

The prevalence estimation showed substantial perception heterogeneity, indicated by the differences between the standard deviations. Regardless of this fact, many participants estimated the prevalence of doping to be considerably higher than the official laboratory data indicated (see Table 2).

**Table 2:** Comparison of athletes from sailing and wrestling concerning their prevalence estimations as well as doping attitudes (PEAS-S), moral perspective (DMDS-S), and resistance against doping (DSRES).

**Analysis section**		**Sailing**	**Wrestling**	**Mann-Whitney test**
		***M*** **(*SD*)**	***M*** **(*SD*)**	
		**[*Mdn*, *MAD*]**	**[*Mdn*, *MAD*]**	
1	International elite sports overall	39.1 % (18.6)	25.5 % (20.0)	*U* = 148.50, *z* = −2.48, *p* = 0.013, *r* = 0.43 [95% CI: 0.19, 0.66]
		[40.0, 10.0]	[22.5, 12.5]	
	National elite sports overall	25.8 % (14.9)	11.8 % (19.1)	*U* = 103.00, *z* = −3.50, *p* < 0.001, *r* = 0.60 [95% CI: 0.33, 0.78]
		[25.0, 9.0]	[5.0, 4.0]	
	International elite sports own sports	9.6 % (9.3)	17.3 % (14.8)	*U* = 177.00, *z* = −1.86, *p* = 0.064, *r* = 0.32 [95% CI: −0.59, 0.01]
		[5.0, 4.0]	[16.5, 7.5]	
	National elite sports own sports	5.4% (8.26)	7.3 % (16.3)	*U* = 245.00, *z* = −0.34, *p* = 0.734
		[2.0, 2.0]	[1.0, 1.0]	
2	PEAS-S (Sum)	13.31 (4.05)	13.24 (4.86)	*U* = 414.50, *z* = −0.41, *p* =0.680
		[12.50, 3.50]	[12.00, 2.50]	
		Range: 8–20	Range: 8–28	
3	DMDS-S	2.40 (0.70)	2.40 (0.74)	*U* = 439.00, *z* = −0.05, *p* =0.964
		[2.50, 0.50]	[2.50, 0.50]	
4	DSRES	4.33 (0.80)	4.55 (0.88)	*U* = 305.50, *z* = −2.15, *p* = 0.031, *r* = 0.31 [95% CI: −0.55, −0.02]
		[4.67, 0.33]	[5.00, 0.00]	

Comparing the two types of sports, sailors perceived doping as more widespread in international and national elite sports in general (see Table 2). The heterogeneity of the participants' perception was apparent in the confidence interval of the effect sizes, which varied between a small to strong effect (international) and a medium to strong effect (national elite sports; Cohen, 1988). There were no differences between sailors and wrestlers concerning the perception of the prevalence of doping in their sport.

Generally, the participants indicated a strongly rejective attitude toward doping (*M* = 13.27, *SD* = 4.49, *Mdn* = 12.00, *MAD* = 3.00). Nevertheless, the participants' attitudes showed a heterogeneous picture and included athletes who indicated solely a slightly rejective doping attitude. No statistical difference emerged between sailors and wrestlers (see Table 2, analysis Section Research Questions).

Participants generally indicated a low willingness to morally disengage in doping concerns (*M* = 2.40, *SD* = 0.72, *Mdn* = 2.50, *MAD* = 0.50). This tendency was independent of the type of sports participants competed in (see Table 2, analysis Section Methods).

Considered first in general and independent of sport, most participants indicated to be very confident to resist doping temptations (*M* = 4.46, *SD* = 0.85, *Mdn* = 4.83, *MAD* = 0.17). Comparing the two types of sports, wrestlers indicated to be even more resistant than sailors, including a medium effect size (Cohen, 1988; see Table 2, analysis Section Results).

**Figure 1:**
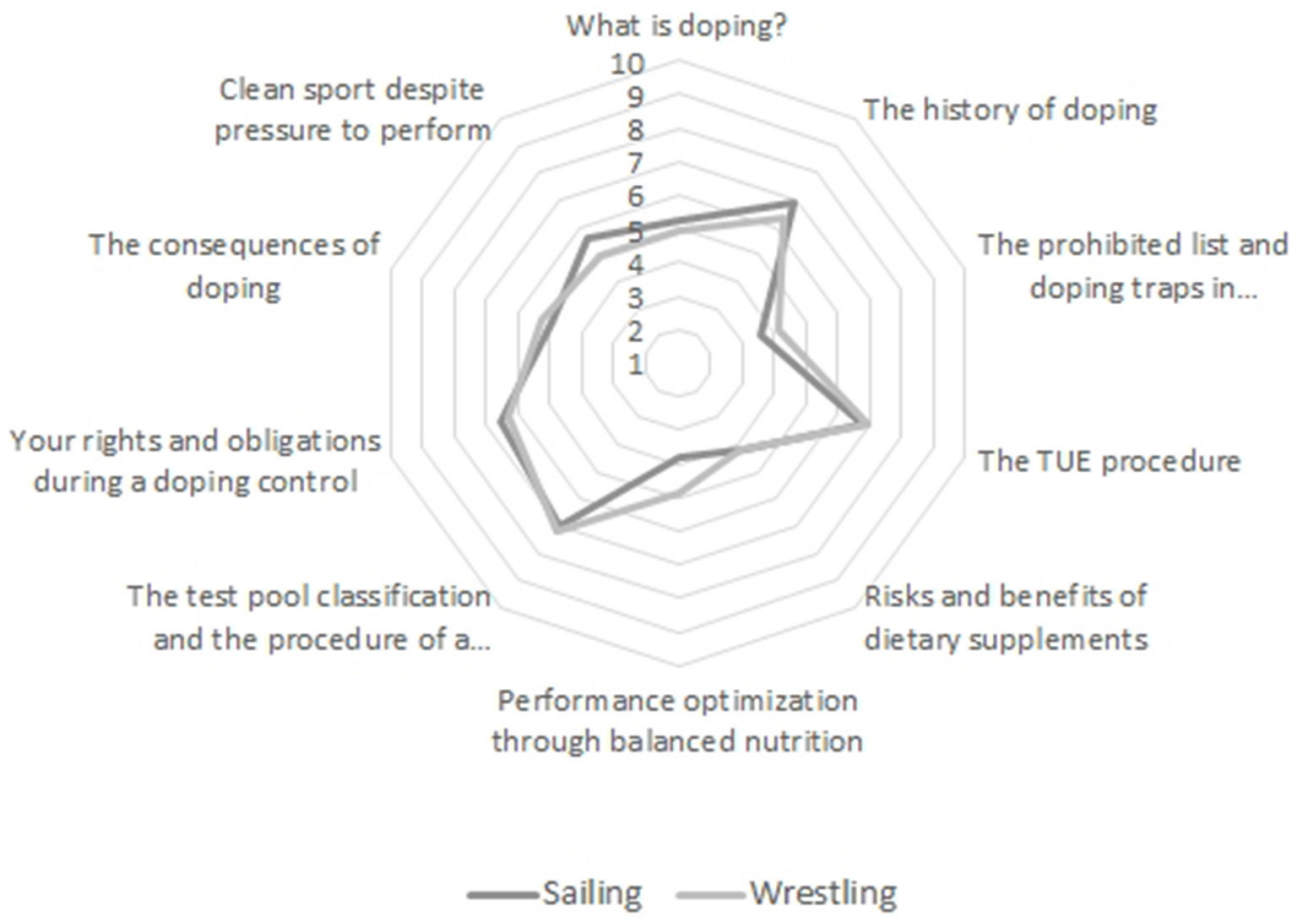
Content preferences regarding German doping prevention program *Together Against Doping*.

No sport-specific profile emerged concerning the topics participants would like to address in an anti-doping measure (see Figure 1, the significance of the Mann-Whitney test comparing sailing and wrestling ranged from *p* = 0.160 to *p* = 0.811). Regarding six of the 10 contents offered, participants exploited the full range of rank options: a topic rated as most important by one participant was rated as the least important by another.

**Figure 2:**
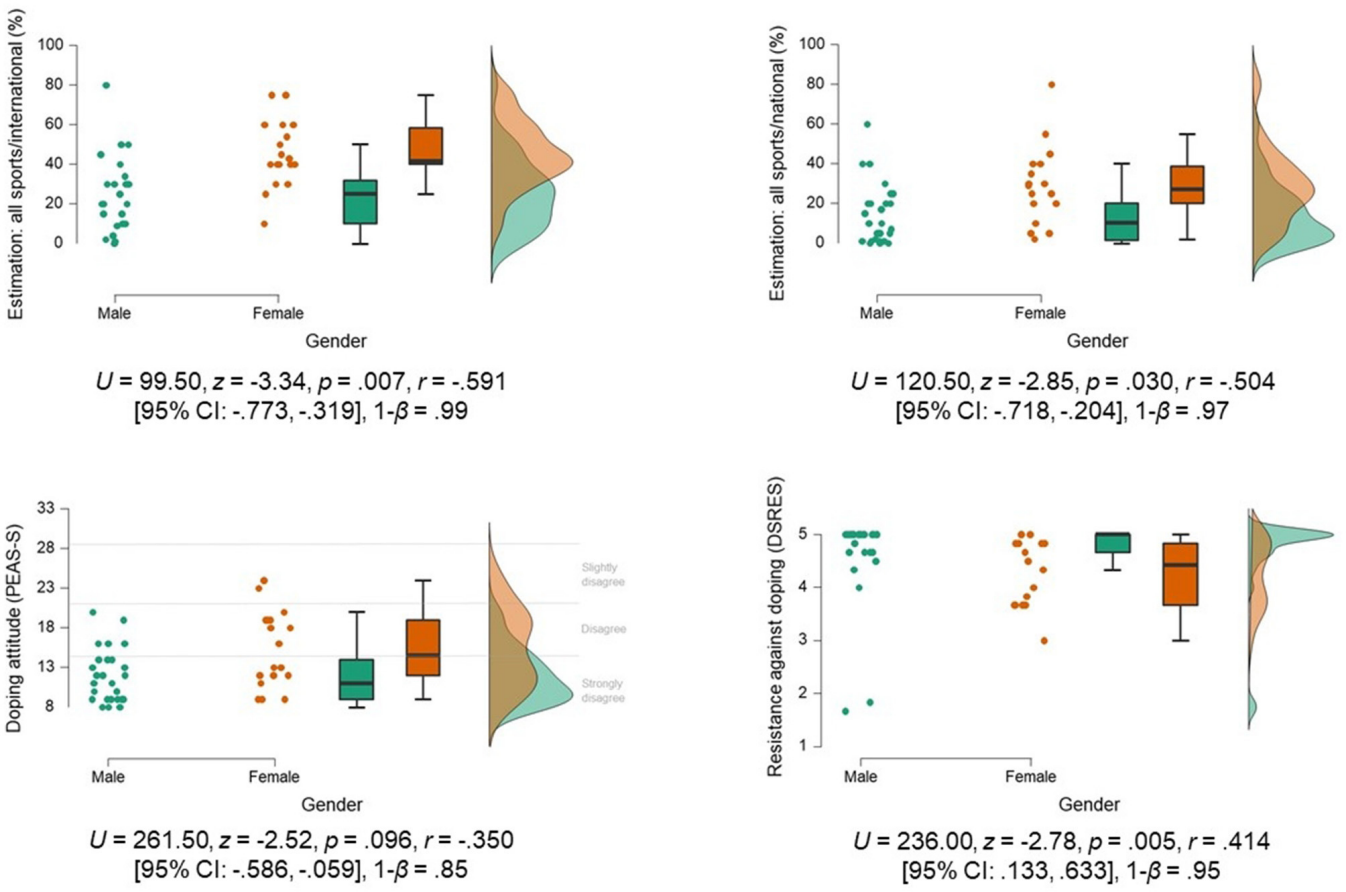
Results of the exploratory data analysis comparing male and female athletes (significance values were adjusted by Bonferroni-Holm procedure for multiple tests).

Deviations and heterogeneity of the data suggest the existence of subgroups beyond a differentiation by type of sports. Since the literature points to doping-related gender differences (Gleaves et al., 2021), a subsequent exploratory data analysis was conducted comparing male and female participants irrespective of the type of sport. Considerable differences can be seen in Figure 2. The analyses showed that young female elite athletes assumed a greater prevalence of doping in national and international sports in general. In addition, their attitudes toward doping were less negative, and they were less confident to resist doping temptations.

## Discussion

Generally, the study cannot identify sport-specific differences for a subsequent prevention planning tailored to these sports within the setting of young elite sports. One must keep in mind that the prevalence logic regarding a high and a low risk for doping was transferred from sports in general to young elite sports. Due to a lack of comparative data from senior elite sports, we cannot assume that sport-specific differences become apparent in the course of the career and socialization in a type of sport with a low or high risk for doping. Instead, all young elite athletes who participated indicate a good baseline for doping prevention. Thus, no indicators can be found why athletes from a sport with a high risk for doping should be prioritized in education in this age group (cf. World Anti-Doping Agency, 2021b).

Athletes perceive doping to be more widespread than laboratory data suggest. Their data support studies that have explicitly dealt with the determination of doping prevalence and assume a significantly higher prevalence of doping (Gleaves et al., 2021). Comparable to coaches' perception (Pöppel and Büsch, 2019), athletes perceive foreign countries to be more *doping-polluted*. Thus, athletes have the impression that cleaner competitions are more likely to occur in the national setting than in the international setting. It is noticeable that there is a more substantial discrepancy in how athletes perceive the situation within their sport. Although athletes should be more familiar with the situation in their sport, one can assume that questions about the prevalence of doping in one's sport trigger the fear of negative conclusions. The data suggest that athletes expect more doped opponents in the international arena. Therefore, the national space should be a good starting point for constructive doping prevention to protect clean athletes early (cf. Petróczi et al., 2021).

Regardless of the type of sports, both groups of athletes appear to be in a favorable starting position, with a negative attitude toward doping. This critical attitude is a typical phenomenon regarding self-reported doping attitudes (e.g., Vargo et al., 2015; Pöppel and Büsch, 2019). However, the heterogeneity of data regarding doping attitudes (see Figure 2) indicate that individual athletes with a slightly negative attitude perish in the group analysis. In particular, for these individuals, doping prevention must be tailored to their needs and needs to address issues, which supports athletes to develop a more reluctant attitude.

Overall, athletes reject morally disengaging behavior. Again, this result is independent of the type of sports. Nevertheless, athletes indicate room for improvement regarding a moral consideration of doping. Individual items allow for external attribution when dealing with doping behavior (e.g., pressure from team members). Therefore, tendencies to relativize one's misconduct should be considered early. In this context, dilemma discussions (e.g., Elbe and Brand, 2016) could be helpful for a critical reflection. The psychometric properties of the assessment of moral disengagement (DMDS-S) are considerably lower in this sample than in the methodological paper that introduces the scale (Cronbach's α = 0.86 and 0.89; Boardley et al., 2018). Therefore, these results need to be interpreted with caution. Considering the width of the confidence interval regarding the effect sizes of the measurement of perceived resistance against doping temptations (DSRES), these results need to be interpreted with caution too (see Table 2, analysis Section Results). Athletes from the type of sports in which doping appears to be more widespread express higher confidence to resist doping temptations. As with the other scales, there might be a tendency for socially desirable responses (e.g., Gucciardi et al., 2010; Petróczi and Nepusz, 2011). Thus, data support the need to integrate indirect measures in doping prevention research (Petróczi, 2016).

Finally, athletes indicate no sport-specific differences concerning doping prevention topics. Regardless of the type of sports, the spider web figure (see Figure 2) shows that none of the topics was considered particularly interesting or uninteresting. All topics ranked in the middle, with a comparatively high degree of heterogeneity in terms of ranking. Even topics that address the desire to increase performance in sport in a constructive way (e.g., healthy nutrition) do not stand out as being highly valued. It is reasonable to consider preferences at a smaller group or individual level. In addition, the present wording of the topics might be too unspecific and thus does not provide enough clues for a clear expression of interest.

The findings of this study indicate that one should consider additional aspects in young elite sports concerning the doping-specific background of young athletes. Clustering by sport is in line with the usual approach when doping prevention is planned in consultation with sports federations. The findings of this study suggest that additional characteristics need to be considered more in young elite sports than the characteristics of a type of sports when designing tailored doping prevention. In line with the review on doping prevalence by Gleaves et al. (2021), the gender of the athletes should be considered as a control variable. The data show that young women indicate a more vulnerable baseline for doping than young men regarding their doping attitudes and a comparably lower confidence to resist doping temptations. These results should be considered in doping prevention specifically. Thus, athletes should profit from a more individualized approach, which considers gender-specifics.

Furthermore, doping prevention should be expanded in the sense of a modular system from which athletes can individually select topics. The description of topics should be more specific and offered in smaller steps than within the *Together Against Doping* program (German Nationale Anti-Doping Agentur, 2020). Expanding the implementation of apps or internet-based prevention components could supplement group measures to increase efficiency. Overall, evaluating (modified) doping prevention is necessary (e.g., Boardley et al., 2021).

### Limitations

Methodological limitations concerning certain aspects of this research need to be acknowledged. As we had access to all young athletes involved in German sailing and wrestling talent development programs and thus a high-quality sample, we did not perform a preliminary power analysis to specify the optimal sample size. In order to assess the meaningfulness of results in this small sample and to better evaluate the explanatory power of effect sizes, a sensitivity analysis was subsequently calculated. According to the analysis, an effect size of *r* = 0.44 is needed to strengthen the significance of the results. If we focus on the prevalence estimates, this effect size is exceeded in comparing national elite sports (*r* = 0.60) and approximately achieved in comparing international sports (*r* = 0.43). These values strengthen the significance of the different prevalence estimates in national and international sports between wrestlers and sailors, focusing on elite sports in general. The self-reported resistance against doping fell below this value (*r* = 0.31). However, the upper level of the confidence interval exceeds the effect size according to the sensitivity analysis (*r* = 0.44, see Table 2, analysis Section Results). Generally, the sensitivity analysis indicates the significance of the results and the robustness of the sample examined.

Furthermore, assessing doping-related characteristics *via* self-report enhances the probability of response bias (e.g., Gucciardi et al., 2010). The actual values of the variables might be less favorable than reported by the athletes. Future studies should integrate indirect measures and enable a more individualized view with more robust procedures while protecting the athletes' anonymity and should add a gender-specific perspective. Therefore, the logic in doping prevention should not be one size fits all, but every athlete counts.

## Data Availability Statement

The datasets presented in this article are not readily available because, the anonymity of participants is to protect. Requests to access the datasets should be directed to Katharina Pöppel, katharina.poeppel@uol.de.

## Ethics Statement

The studies involving human participants were reviewed and approved by Carl von Ossietzky University of Oldenburg, Germany, Local Ethics Committee. Written informed consent to participate in this study was provided by the participants' legal guardian/next of kin.

## Author Contributions

KP designed the survey in consultation with DB, obtained ethical approval for the intervention, conducted the data coding, transcription, and analysis. DB initiated contact with the federations. All authors contributed to the study design, writing and revision process, and approved the final manuscript.

## Conflict of Interest

The authors declare that the research was conducted in the absence of any commercial or financial relationships that could be construed as a potential conflict of interest.

## Publisher's Note

All claims expressed in this article are solely those of the authors and do not necessarily represent those of their affiliated organizations, or those of the publisher, the editors and the reviewers. Any product that may be evaluated in this article, or claim that may be made by its manufacturer, is not guaranteed or endorsed by the publisher.
